# The importance of synchronicity in the management of colorectal peritoneal metastases with cytoreductive surgery and hyperthermic intraperitoneal chemotherapy

**DOI:** 10.1186/s12957-020-1784-4

**Published:** 2020-01-13

**Authors:** Jolene Si Min Wong, Grace Hwei Ching Tan, Claramae Shulyn Chia, Johnny Ong, Wai Yee Ng, Melissa Ching Ching Teo

**Affiliations:** Division of Surgical Oncology, National Cancer Centre Singapore, 9 Hospital Drive, Singapore, 169610 Singapore

**Keywords:** Colorectal peritoneal metastases, Synchronous, Metachronous

## Abstract

**Background:**

Colorectal peritoneal metastases (CPM) occur in up to 13% of patients with colorectal cancer, presenting either synchronously or metachronously. Cytoreductive surgery and hyperthermic intraperitoneal chemotherapy (CRS and HIPEC) have been increasingly utilised for selected CPM patients with favourable outcomes, though its benefits may differ for synchronous (s-CPM) and metachronous CPM (m-CPM).

**Methods:**

A retrospective analysis of CPM patients treated with CRS and HIPEC at the National Cancer Centre Singapore over 15 years was performed. In the s-CPM group, CPM was diagnosed at primary presentation with CRS and HIPEC performed at the time of or within 6 months from primary surgery. In the m-CPM group, patients developed CPM > 6 months after primary curative surgery.

**Results:**

One hundred two patients with CPM were treated with CRS and HIPEC. Twenty (19.6%) patients had s-CPM and 82 (80.4%) had m-CPM. Recurrences occurred in 45% of s-CPM and in 54% of m-CPM (*p* = 0.619). Median overall survival was significantly prolonged in patients with m-CPM (45.2 versus 26.9 months, *p* = 0.025). In a subset of m-CPM patients with limited PCI in whom ICU stay was not required, a survival advantage was seen (*p* = 0.031).

**Conclusion:**

A survival advantage was seen a subset of m-CPM patients, possibly representing differences in disease biology.

## Introduction

Peritoneal metastases (PM) occur in up to 20% of patients with stage IV colorectal cancer (CRC) [1]. The peritoneum has been recognised as a site of metastases that is distinct from its pulmonary and hepatic counterparts where a hypoxic environment may hinder the penetration of systemic chemotherapeutic agents [2]. In an attempt to improve local-regional delivery of cytotoxic drugs, Spratt and Sugarbaker developed the concept of cytoreductive surgery (CRS) and hyperthermic intra-peritoneal chemotherapy (HIPEC) [3, 4]. At present, CRS and HIPEC when performed for selected patients with peritoneal-only metastases boost a median overall survival (OS) of 30 months [5], superior to modern chemotherapy regimens (reported median OS of 12 to 24 months) [6].

In CRC with hepatic metastases, it has been proposed that a synchronous or metachronous presentation implied differing tumour biologies [7]. Synchronous liver disease has been associated with a more aggressive clinical picture, with patients experiencing poorer survival outcomes when compared with their metachronous counterparts [8]. Amongst colorectal peritoneal metastases (CPM), the reported proportion of synchronous metastases (s-CPM) was 60%; median OS was 7 months in s-CPM patients while the metachronous group saw a median of 12 months survival from time of diagnosis of CPM in the era of palliative systemic therapy [9]. To date, the role of CRS and HIPEC in the management of CPM has been evaluated in two large randomised control trials (RCTs) and multiple retrospective series [5, 10–12] and none, however, attempted to differentiate between s-CPM and m-CPM.

As such, our study aims to compare survival and recurrence outcomes of patients with s-CPM and m-CPM in the context of CRS and HIPEC. We believe that discussing their outcomes independently may shed light on their possibly distinct biology and is crucial in any management algorithm undertaken.

## Materials and methods

### Patient selection and data

The current study was performed in a single tertiary institution. Data was retrieved from a prospectively maintained database of patients treated with CRS and HIPEC for CPM between January 2003 and January 2018.

Our primary endpoints were overall survival (OS) and progression-free survival (PFS). Clinical characteristics, operative data, and 30-day morbidity and mortality were also evaluated.

The study was conducted with the approval of the Centralized Institutional Review Board of Singapore Health Services.

### Key definitions

Patients were classified into two groups for comparison:

1. Synchronous CPM (s-CPM) – CPM present at the time of the first diagnosis for which CRS and HIPEC were performed within 6 months of diagnosis
2. Metachronous CPM (m-CPM) – CPM was *not* present at the time of the first diagnosis of colorectal cancer but detected at subsequent follow-up and for which CRS and HIPEC were performed

In both groups, OS was defined as time in months, between CRS and HIPEC to date of last follow-up or death, while PFS was defined as the time interval from the date of CRS and HIPEC to the date of detection of recurrent disease.

The Peritoneal Cancer Index (PCI) score as described by Sugarbaker was used to describe the extent of disease [13]. The completeness of cytoreduction (CC) score was utilised to measure the amount of residual disease [14], with CC-0/1 considered as optimal cytoreduction.

### Selection of patients for pre-operative systemic treatment

The decision for upfront CRS and HIPEC versus neoadjuvant treatment prior to surgery was guided by a multi-disciplinary tumour board comprising of surgical, medical and radiation oncologists, pathologists and radiologists. Patient fitness and preference; tumour biology, inferred by PCI score; disease-free interval; primary tumour characteristics (grade of differentiation; histological features, e.g. mucinous; signet ring cell; molecular status; e.g. RAS; BRAF mutations); and confidence of surgeon to achieve a CC0 resection were all considerations in the selection of patients for pre-operative systemic therapy.

### CRS and HIPEC and follow-up

The CRS and HIPEC procedure performed at our institution was as previously described [15, 16] and involved the removal of all macroscopic peritoneal disease to achieve complete cytoreduction, with the subsequent administration of HIPEC. A closed technique for HIPEC was adopted. For CPM, mitomycin C was administered (dose of 12.5 mg/m^2^ for males and 10 mg/m^2^ for females) with 4 L of peritoneal dialysis solution at 41–42 °C over a duration of 60 min. A hyperthermia pump was used during the study duration to deliver the intraperitoneal chemotherapy agent via a single inflow catheter, and drainage was via four intra-abdominal drains.

Post-operatively, patients were transferred to the surgical intensive care unit (SICU) or high-dependency unit for monitoring. All intra- and post-operative complications were recorded and graded based on the Clavien-Dindo classification [17].

During follow-up, patients were reviewed at 3 monthly intervals during which a full physical examination and tumour markers were taken. A computed tomography (CT) scan of the chest-abdomen and pelvis was performed 6 monthly for the first 2 years post CRS-HIPEC and then yearly thereafter. Details of recurrences, if any, were recorded.

### Statistical analysis

Differences in demographics and clinical characteristics of the patients and recurrent patients were assessed between the two groups (1) s-CPM and (2) m-CPM by using Fisher’s exact test for categorical variables and two group *t* tests for numeric variables. Wilcoxon rank-sum test was used if the distributions were skewed for numeric variables. Survival functions were estimated using Kaplan-Meier method, and log-rank test was used to evaluate the differences between the two groups. Univariate Cox regression was applied to investigate potential factors on the risk of the event of death and recurrence. Variables with *p* value less than 0.10 in the univariate analysis were included in the multivariate Cox regression model. The model was built using backward selection. Variables with *p* value < 0.05 would be in the final model. Cox proportionality assumption was assessed by using an overall test on Schoenfeld residuals.

A two-sided *p* value of < 0.05 was considered statistically significant. All analyses were performed using Stata version 12.0.

## Results

### Patient and tumour characteristics

A total of 102 patients with CPM underwent CRS and HIPEC from January 2003 to January 2018. There were 20 (20%) patients with s-CPM and 82 (80%) with m-CPM. Ninety-seven percent of all patients with CPM and all with s-CPM had locally advanced, i.e. T3/T4 primary tumours. Pre-operative CEA levels was significantly higher in the s-CPM patients (*p* = 0.043). Other baseline clinical-pathological characteristics are as described in Table 1.

**Table 1:** Demographics and clinical characteristics of CPM patients undergoing CRS and HIPEC

	All CPM (*n* = 102)	s-CPM (*n* = 20)	m-CPM (*n* = 82)	*p* value
Patient characteristics				
Age, years	54.0 (24–78)	49 (24–72)	55 (30–78)	0.132
Gender				1.000
Male	39 (38.2%)	8 (40%)	31 (37.8%)	
Female	63 (61.8%)	12 (60%)	51 (62.2%)	
Race				0.004
Chinese	85 (82.5%)	12 (60.0%)	73 (89.0%)	
Others	17 (17.5%)	8 (40.0%)	9 (11.0%)	
ECOG status				0.350
0/1	94 (92.2%)	20 (100.0%)	74 (90.2%)	
2	8 (7.8%)	0 (0.0%)	8 (9.8%)	
Tumour characteristics				
T-stage (primary tumour)				0.502
T1–2	3 (2.9%)	0 (0.0%)	3 (3.7%)	
T3–4	99 (97.1%)	20 (100%)	79 (96.3%)	
N-stage (primary tumour)				0.15
N0–1	63 (61.8%)	11 (55%)	52 (63.4%)	
N2	31 (30.4%)	6 (30%)	25 (30.4%)	
Unknown	8 (7.8%)	3 (15%)	5 (6.2%)	
Tumour differentiation (primary tumour)				1.02
Well	5 (5%)	1(5%)	4 (4.8%)	
Moderate	60 (58.8%)	10 (50%)	50 (61%)	
Poor	31 (30.4%)	7(35%)	24 (29.3%)	
Unknown	6 (5.8%)	2 (10%)	4 (4.9%)	
Histology (primary tumour)				0.51
Adenocarcinoma	74 (72.5%)	13 (65.0%)	61 (74.5%)	
Mucinous	23 (22.5%)	6 (30.0%)	17 (20.7%)	
Signet ring cell	2 (2%)	0 (0.0%)	2 (2.4%)	
Mixed	2 (2%)	1 (5.0%)	1 (1.2%)	
Others	1 (1%)	0 (0.0%)	1 (1.2%)	
Sidedness of Tumour (primary tumour)				0.54
Right	32 (31.4%)	7 (35%)	25 (30.5%)	
Left	51 (50%)	10 (50%)	41 (50%)	
Rectum	15 (14.7%)	1 (5%)	14 (17%)	
Unknown	4 (3.9%)	2 (10%)	2 (2.5%)	
Pre-operative CEA levels, mean (μG/l)	32.3	43.1 (1.1 - 501)	29.8 (0.5 – 441)	0.043
Intra-operative				
PCI score, median (range)	7 (0–27)	9 (3–27)	5 (0–24)	0.12
PCI score				0.11
< 10	55 (54%)	10 (50%)	45 (54.9%)	
10–20	31 (30.3%)	4 (20%)	27 (32.9%)	
> 20	5 (5%)	3 (15%)	2 (3.8%)	
Unknown	11 (10.7%)	3 (15%)	8 (8.4%)	
CC-score				0.352
CC0	101 (98.1%)	19 (95.0%)	82 (100%)	
CC1	1 (1.0%)	1 (5.0%)	0 (0.0%)	

*PCI* Peritoneal Cancer Index, *CC-score* Completeness of Cytoreduction Score

### Surgery and peri-operative outcomes

In the s-CPM group, 75% (*n* = 15) received neo-adjuvant chemotherapy or chemoradiation therapy before CRS and HIPEC were performed. Agents used were mainly 5-fluorouracil (FU) based with the addition of oxaliplatin or irinotecan. Targeted agents were used at the discretion of the medical oncologists after consideration of general response and molecular status. In the m-CPM group, the median time between surgery for the primary tumour and the development of metachronous metastases was 21.7 months (range 7.9–186.2). Eleven (13%) patients received neoadjuvant chemotherapy prior to CRS and HIPEC.

The CRS and HIPEC duration was 390 min (range 245–855) in s-CPM and was 415 min (range 200–960) in m-CPM (*p* = 0.618). There were no differences in terms of intra-operative blood loss (1200 ml versus 1003 ml).

Overall, the median PCI score was 7 (range 0–27). There was no significant difference in the PCI scores when comparing s-CPM and m-CPM (9 versus 5, *p* = 0.12). All patients received complete cytoreduction surgery.

Post CRS and HIPEC, the decision for further adjuvant chemotherapy was discussed at our multi-disciplinary tumour board. Twenty-eight (27.2%) patients received further systemic therapy, of which nine patients were from the s-CPM group and 19 the m-CPM group.

Overall, the median duration of hospital stay was 12 days (range 7–66), and it was 14.5 days (range 7–26) and 11 days (range 7–66) in s-CPM and m-CPM patients, respectively (*p* = 0.198). Post-operative complications occurred in 47% (*n* = 48) of patients with no difference between the two groups. Majority of patients (86%) suffered Clavein-Dindo grade 1 or 2 complications. Of the 15 patients who required invasive intervention (i.e. grade 3 or 4 complications), four patients suffered pleural effusions necessitating chest tube insertion; two patients had post-operative bleeding needing re-laparotomy; and two patients had a ureteric leak requiring insertion of percutaneous nephrostomy (PCN) tubes, with three patients having intra-abdominal collections, three patients with anastomotic leak requiring insertion of abdominal drain and one patient with acute retention of urine requiring the insertion of urinary catheter. There was no in-hospital mortality.

### Recurrence outcomes

In total, 54 (52.4%) patients developed recurrences after CRS and HIPEC: nine (*n* = 9/20, 45%) from the s-CPM group and 45 (*n* = 45/82, 54.9%) from the m-CPM group (*p* = 0.619). Median time to recurrence was 9.5 months (range 0.9–33.7). It was 13.1 (range 2.6–17.8) and 9.5 (range 0.9–33.7) months respectively in s-CPM and m-CPM groups (*p* = 0.917). The pattern of recurrence was as described in Table 2.

**Table 2:** Pattern of recurrence post CRS and HIPEC surgery for s-CPM and m-CPM patients

Pattern of recurrence	All recurrent CPM (*n* = 54)	s-CPM (*n* = 9)	m-CPM (*n* = 45)	*p* values
Peritoneum	33 (61.1%)	7 (77.8%)	26 (57.8%)	0.45
Peritoneum only	15	5	10	
Peritoneum and lung	5	–	5	
Peritoneum and liver	3	–	3	
Peritoneum and anastomosis	6	2	4	
Peritoneum, liver, and lung	1	–	1	
Peritoneum, bone, and abdominal wall	1	–	1	
Peritoneum, liver, lung, bone, and abdominal wall	1	–	1	
Peritoneal, liver, lung, and spleen	1	–	1	
Lung	8 (14.8%)	1 (11.1%)	7 (15.5%)	1.00
Lung only	4	1	3	
Lung and anastomosis	2	–	2	
Lung, bone, and abdominal wall	1	–	1	
Lung, liver, bone, and CNS	1	–	1	
Liver	6 (11.1%)	1 (11.1%)	5 (11.2%)	0.453
Liver only	4	1	3	
Liver and CNS	1	–	1	
Liver and kidney	1	–	1	
Others	7 (13%)	–	7 (15.5%)	0.112

**Fig. 1:**
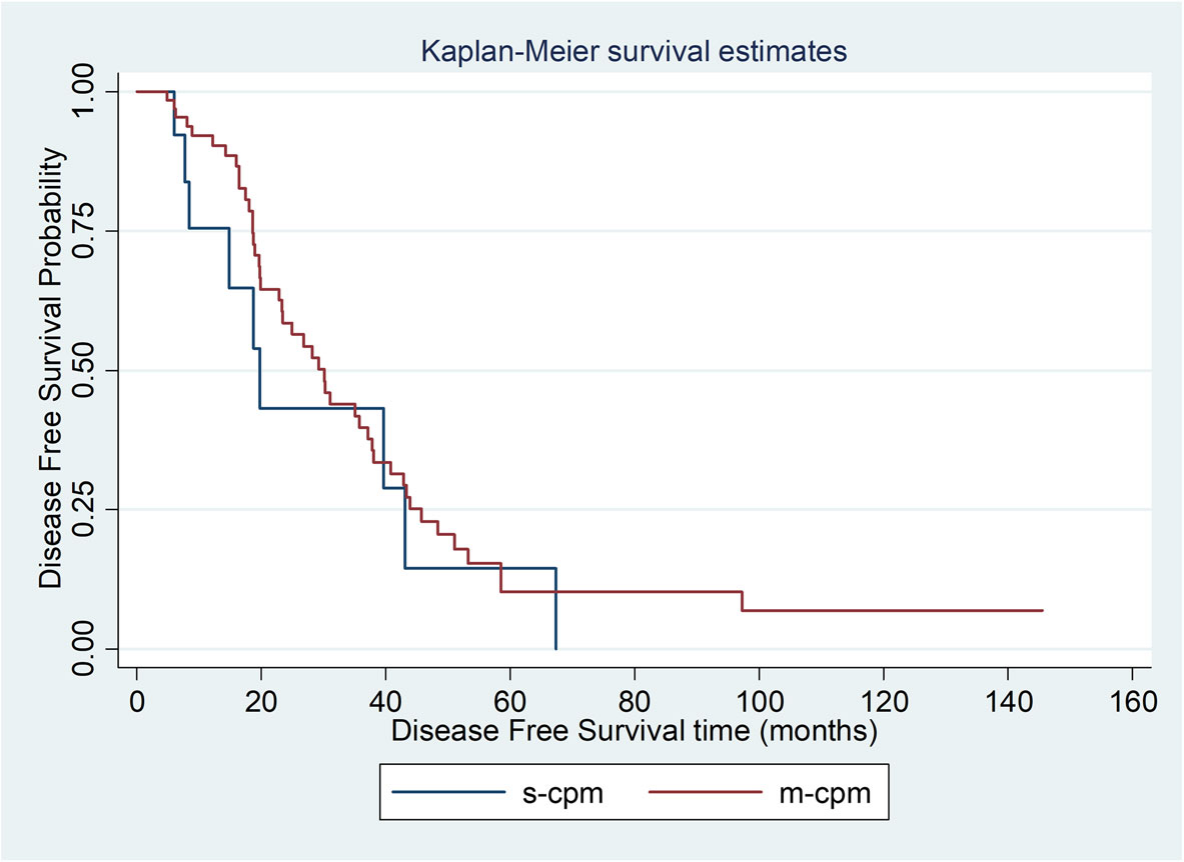
Progression-free survival for s-CPM versus m-CPM post CRS and HIPEC

There was however no significant difference in PFS between s-CPM and m-CPM groups (*p* = 0.356; Table 3, Fig. 1). Median PFS was 19.7 months (range 7.7–43.2) in s-CPM and 30.2 months (range 19.8–37.8) in m-CPM.

**Table 3:** Overall survival (OS) and progression-free survival (PFS) for s-CPM and m-CPM patients post CRS and HIPEC

	No. of events/no. of patient	Median OS, (95% CI)	1 year rate, % (95% CI)	3 year rate, % (95% CI)	5 year rate,% (95% CI)	*p* value
Overall survival						
s-CPM	9/20	26.9 (9.8–44.5)	77.0 (43.2–92.2)	27.5 (4.5–58.4)	13.8 (0.8–44.7)	0.025
m-CPM	25/82	45.2 (30.2–54.3)	92.1 (81.3–96.0)	62.0 (45.1–74.6)	31.2 (15.1–48.4)	
Progression-free survival						
s-CPM	9/20	19.7 (7.7–43.2)	75.5 (41.6–91.4)	28.8 (5.2–59.1)	0	0.356
m-CPM	45/82	30.2 (19.8–37.8)	92.2 (82.3–96.7)	39.8 (26.2–52.9)	10.2 (3.5–21.5)

### Survival outcomes

**Fig. 2:**
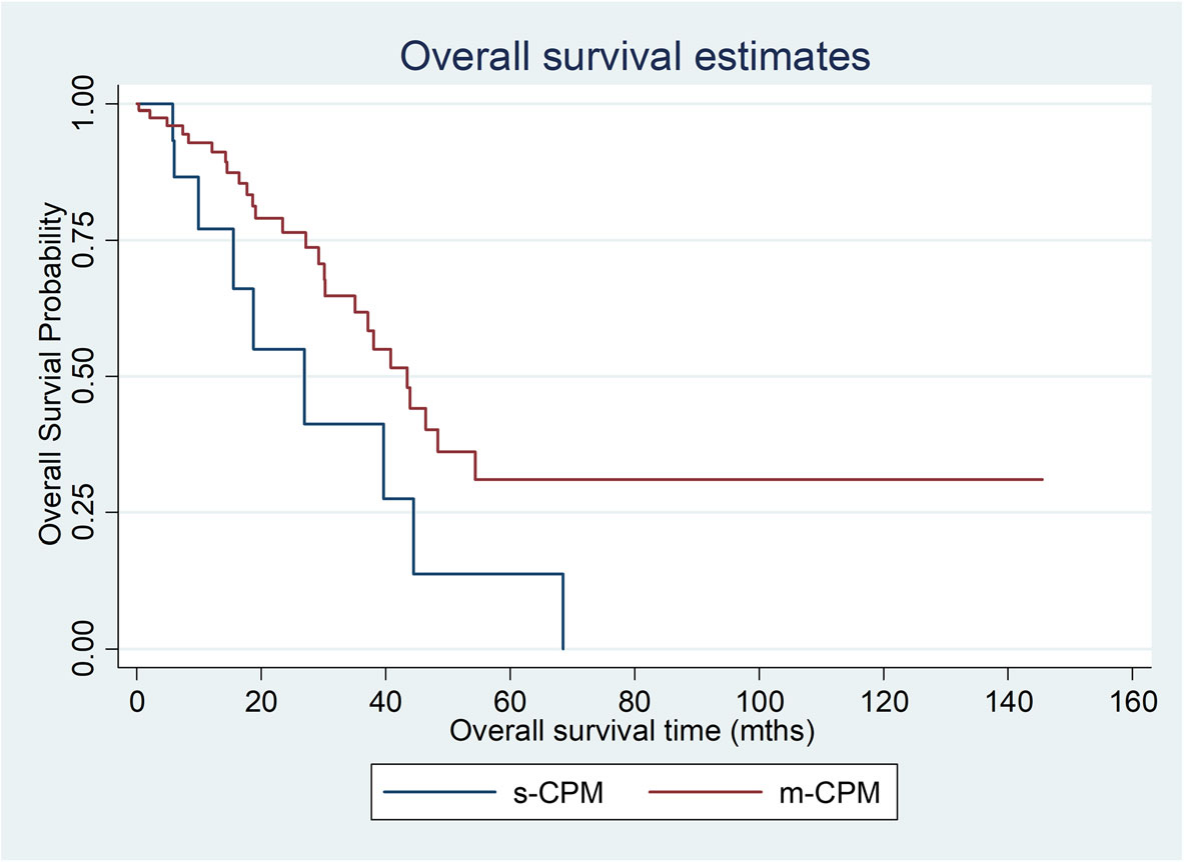
Overall survival for s-CPM versus m-CPM post CRS and HIPEC

Overall median OS in all CPM patients was 40.6 months. Comparing s-CPM and m-CPM, a significant difference was found in OS outcomes: 26.9 months (range 9.8–44.5) in the former and 45.2 months (range 30.2–54.3) in the latter (*p* = 0.025). One-, 3-, and 5-year OS is as illustrated in Table 3 and Fig. 2.

In the univariate analysis, synchronous disease, need for ICU stay, PCI > 12, older age and longer duration of CRS and HIPEC were significant predictors for overall survival. On multivariate analysis, only ICU stay, PCI > 12, older age, and longer duration of CRS and HIPEC remained significant (Table 4).

**Table 4:** Uni- and multivariable comparison of OS of CPM after CRS and HIPEC

Variable	Univariate hazard ratio (95% CI)	*p* value	Multivariate hazard ratio (95% CI)	*p* value
m-CPM versus s-CPM	0.45 (0.21–0.97)	0.042	0.92 (0.33–2.61)	0.880
Age	0.96 (0.94–0.99)	0.016	0.94 (0.90–0.98)	0.004
Duration of CRS and HIPEC	1.003 (1.001–1.005)	0.004	0.99 (0.990–0.999)	0.014
ICU stay	1.14 (0.99–1.31)	0.065	3.16 (1.47–6.76)	0.003
Median length of stay	1.03 (0.99–1.06)	0.052	1.06 (1.01–1.11)	0.009
PCI score > 12	1.11 (1.06–1.16)	< 0.001	1.10 (1.02–1.18)	0.011

On subgroup analysis adjusted for ICU stay, it was found that in patients who did not require ICU stay, s-CPM was associated with poorer OS (*p* = 0.034). In addition, in the subset of m-CPM patients with PCI < 12, a more significant survival advantage was seen (Table 5).

**Table 5:** Subgroup analysis of OS outcomes based on ICU stay and PCI score

Variable	Univariate hazard ratio (95% CI)	*p* value	Multivariate hazard ratio (95% CI)	*p* value
CRS and HIPEC patients without ICU stay				
m-CPM versus s-CPM	0.27 (0.06–1.15)	0.077	0.11 (0.02–0.85)	0.034
Age	0.96 (0.91–1.01)	0.112	0.99 (0.94–1.05)	0.759
PCI score	1.14 (1.02–1.27)	0.020	1.14 (0.99–1.30)	0.063
Duration of CRS and HIPEC	1.00 (0.99–1.01)	0.145	1.01 (0.99–1.02)	0.215
PCI < 12				
m-CPM versus s-CPM	0.18 (0.05–0.67)	0.011	0.19 (0.04–0.86)	0.031
Age	0.95 (0.89–1.02)	0.149	0.98 (0.92–1.03)	0.399
Duration of CRS and HIPEC	1.002 (0.999–1.006)	0.244	0.997 (0.990–1.003)	0.316
SICU stay	1.91 (0.93–3.91)	0.078	3.95 (0.74–21.25)	0.109

## Discussion

The tumour cell entrapment hypothesis has been postulated to be the mechanism behind the occurrence of peritoneal disease [18]. Locally advanced primary CRC result in the spillage and dissemination of tumour cells into the peritoneal cavity. Subsequent implantation then leads to the development of CPM. Introduced in the late twentieth century, CRS and HIPEC aim to remove all macroscopic and microscopic peritoneal disease, in the hope of improving penetration of cytotoxic agents into the peritoneum [3]. In 2003, Verwaal et al. proved the efficacy of CRS and HIPEC over systemic chemotherapy in the management of CPM [10]. This was further supported numerous multi-centre studies and robust meta-analysis on this unique disease entity [2, 19]. In our centre, we have reported median OS for CPM after CRS and HIPEC of 40.9 months, a significant improvement even when compared to modern chemotherapeutic regimes [6].

Both synchronous and metachronous PM are known indications for CRS and HIPEC in CRC. Unlike hepatic, pulmonary and para-aortic lymph node metastases for which synchronous disease have been found to be associated with an aggressive biology and poorer survival [7, 20, 21], data for CPM is scarce. While Jayne et al. reported poorer survival trends in the synchronous disease [9], a recent retrospective analysis by the Dutch group failed to prove a difference in outcomes between the two [22]. With a significantly reduced OS seen in the s-CPM group, our study further compounds the likelihood of poor tumour biology in patients who present with synchronous disease.

At present, rates of s-CPM in published studies range from 20% to 80%. In the PRODIGE 7 trial [11], 30% and 20.5% of its participants had s-CPM in the HIPEC and no HIPEC arms, respectively. The absence of a significant survival advantage in patients who underwent HIPEC (median OS 41.7 months) versus no HIPEC (median OS 41.2 months) challenges to contradict its previously established role in CPM. The Japanese group in an attempt to prove the efficacy of optimal CRS without HIPEC evaluated 78 patients with s-CPM only—a median OS of 33.4 months was reported [23]. The trend towards reduced survival in patients with synchronous disease echoes the findings of our study. As surgical oncologists worldwide scramble to make sense of the findings of the PRODIGE 7, we believe that the concept of synchronicity should be considered to better select for patients that will benefit most from CRS and HIPEC.

Recurrence despite CRS and HIPEC is common, occurring in up to 65% of patients with CPM [24]. In our cohort, 52% of patients suffered recurrent disease—though the pattern of recurrence appears to differ between patients with s-CPM and m-CPM with the former demonstrating a trend towards peritoneal recurrence (Table 2). This again points to the plausibility of differing biology between the two groups. In our m-CPM patients, a long disease-free interval (DFI) of 21.7 months was observed between primary surgery and the first recurrence. It is known that stable disease with chemotherapy and a long DFI often result in improved survival outcomes [25]. As such, a selection bias exists in the metachronous group as potential, and only the ‘better players’ were selected to undergo eventual CRS and HIPEC. This may account for the better OS seen in m-CPM. While no consensus has been reached with regards to the optimal selection of patients for CRS and HIPEC, stringent criteria taking into account disease-free intervals, response to systemic chemotherapy, PCI scores and primary tumour characteristics is paramount to ensure good recurrence and survival outcomes.

The retrospective design and relatively small numbers in this study may have resulted in selection bias as well as a failure to show a significant difference between s-CPM and m-CPM groups in the multi-variate analysis. Though subgroup analysis pointing to a possible trend improves OS in the m-CPM group after accounting for the PCI score and ICU stay, further prospective studies with larger sample sizes will be necessary to further elucidate the true biological differences between s-CPM and m-CPM.

## Conclusion

In a subset of m-CPM patients with limited peritoneal disease in whom intensive care post-operatively was not required, a survival advantage was seen over the s-CPM group. This may represent differences in disease biology and emphasises the need to approach these patients differently. Further prospective studies are needed to determine the appropriate management of s-CPM versus m-CPM.

## Data Availability

The datasets during and/or analysed during the current study available from the corresponding author on reasonable request.

## References

[1] van Gestel YR, de Hingh IH, van Herk-Sukel MP (2014). Patterns of metachronous metastases after curative treatment of colorectal cancer. Cancer Epidemiol.

[2] Teicher BA, Kowal CD, Kennedy KA, Sartorelli AC (1981). Enhancement by hyperthermia of the in vitro cytotoxicity of mitomycin C toward hypoxic tumor cells. Cancer Res.

[3] Spratt JS, Adcock RA, Muskovin M (1980). Clinical delivery system for intraperitoneal hyperthermic chemotherapy. CancerRes.

[4] Gilly FN, Beaujard A, Glehen O et al. Peritonectomy combined with intraperitoneal chemohyperthermia in abdominal cancer with peritoneal carcinomatosis: phase I-II study. Anticancer Res1999; 19: 2317-2321. 10472351

[5] Elias D, Gilly F, Boutitie F (2010). Peritoneal colorectal carcinomatosis treated with surgery and perioperative intraperitoneal chemotherapy: retrospective analysis of 523 patients from a multicentric French study. J Clin Oncol.

[6] Köhne CH, van Cutsem E, Wils J (2005). Phase III study of weekly high-dose infusional fluorouracil plus folinic acid with or without irinotecan in patients with metastatic colorectal cancer: European Organisation for Research and Treatment of Cancer Gastrointestinal Group Study 40986. J Clin Oncol..

[7] Tan EK, Ooi LL (2010). Colorectal cancer liver metastases - understanding the differences in the management of synchronous and metachronous disease. Ann Acad Med Singapore.

[8] Slesser AA, Georgiou P, Brown G (2013). The tumour biology of synchronous and metachronous colorectal liver metastases: a systematic review. Clin Exp Metastasis.

[9] Jayne DG, Fook S, Loi C, Seow-Choen F (2002). Peritoneal carcinomatosis from colorectal cancer. Br J Surg.

[10] Verwaal VJ, van Ruth S, de Bree E (2003). Randomized trial of cytoreduction and hyperthermic intraperitoneal chemotherapy versus systemic chemotherapy and palliative surgery in patients with peritoneal carcinomatosis of colorectal cancer. J Clin Oncol..

[11] Quenet F, Elias D, Roca L, et al. A UNICANCER phase III trial of hyperthermic intra-peritoneal chemotherapy (HIPEC) for colorectal peritoneal carcinomatosis (PC): PRODIGE 7 (abstract). J Clin Oncol. 2018;36.

[12] Huang CQ, Min Y, Wang SY (2017). Cytoreductive surgery plus hyperthermic intraperitoneal chemotherapy improves survival for peritoneal carcinomatosis from colorectal cancer: a systematic review and meta-analysis of current evidence. Oncotarget.

[13] Sugarbaker PH (1995). Peritonectomy procedures. Ann Surg..

[14] Sugarbaker PH, Jablonsky KA (1995). Prognostic features of 51 colorectal and 130 appendiceal cancer patients with peritoneal carcinomatosis treated by cytoreductive surgery and intraperitoneal chemotherapy. Ann Surg..

[15] Teo MC, Tan GH, Tham CK (2013). Cytoreductive surgery and hyperthermic intraperitoneal chemotherapy in Asian patients: 100 consecutive patients in a single institution. Ann Surg Oncol.

[16] Teo MC, Ching Tan GH, Lim C (2015). Colorectal peritoneal carcinomatosis treated with cytoreductive surgery and hyperthermic intraperitoneal chemotherapy: the experience of a tertiary Asian center. Asian J Surg.

[17] Dindo D, Demartines N, Clavien PA (2004). Classification of surgical complications: a new proposal with evaluation in a cohort of 6336 patients and results of a survey. Ann. Surg..

[18] Sugarbaker PH (2016). Cytoreductive surgery and hyperthermic intraperitoneal chemotherapy in the management of gastrointestinal cancers with peritoneal metastases: progress toward a new standard of care. Cancer Treat Rev.

[19] Mirnezami R, Mehta AM, Chandrakumaran K (2014). Cytoreductive surgery in combination with hyperthermic intraperitoneal chemotherapy improves survival in patients with colorectal peritoneal metastases compared with systemic chemotherapy alone. Br J Cancer.

[20] Wong JS, Tan GH, Teo MC (2016). Management of para-aortic lymph node metastasis in colorectal patients: a systemic review. Surg Oncol.

[21] Cao G, Cheng D, Ye L, et al. Surgical resection of pulmonary metastases from colorectal cancer: 11 years of experiences. PLoS One; 2017 06 18; 12(4):e0175284 10.1371/journal.pone.0175284 PMC5386242 28394911

[22] Hentzen JEKR, Rovers KP, Kuipers H (2019). Impact of synchronous versus metachronous onset of colorectal peritoneal metastases on survival outcomes after cytoreductive surgery (CRS) with hyperthermic intraperitoneal chemotherapy (HIPEC): a multicenter, retrospective, observational study. Ann Surg Oncol.

[23] Shida D, Tsukamoto S, Ochiai H (2018). Long-term outcomes after R0 resection of synchronous peritoneal metastasis from colorectal cancer without cytoreductive surgery or hyperthermic intraperitoneal chemotherapy. Ann Surg Oncol.

[24] Verwaal VJ, Boot H, Aleman BM (2004). Recurrences after peritoneal carcinomatosis of colorectal origin treated by cytoreduction and hyperthermic intraperitoneal chemotherapy: location, treatment, and outcome. Ann Surg Oncol.

[25] Sluiter NR, Rovers KP, Salhi Y (2018). Metachronous peritoneal metastases after adjuvant chemotherapy are associated with poor outcome after cytoreduction and HIPEC. Ann Surg Oncol.

